# PupDB: a database of pupylated proteins

**DOI:** 10.1186/1471-2105-13-40

**Published:** 2012-03-16

**Authors:** Chun-Wei Tung

**Affiliations:** 1 School of Pharmacy, Kaohsiung Medical University, Kaohsiung 807, Taiwan

## Abstract

**Background:**

Prokaryotic ubiquitin-like protein (Pup), the firstly identified post-translational protein modifier in prokaryotes, is an important signal for the selective degradation of proteins. Recently, large-scale proteomics technology has been applied to identify a large number of pupylated proteins. The development of a database for managing pupylated proteins and pupylation sites is important for further analyses.

**Description:**

A database named PupDB is constructed by collecting experimentally identified pupylated proteins and pupylation sites from published studies and integrating the information of pupylated proteins with corresponding structures and functional annotations. PupDB is a web-based database with tools for browses and searches of pupylated proteins and interactive displays of protein structures and pupylation sites.

**Conclusions:**

The structured and searchable database PupDB is expected to provide a useful resource for further analyzing the substrate specificity, identifying pupylated proteins in other organisms and developing computational tools for predicting pupylation sites. PupDB is freely available at http://cwtung.kmu.edu.tw/pupdb.

## Background

Protein-to-protein modifications are essential for regulating protein functions. In eukaryotes, ubiquitylation involved in numerous regulatory functions such as protein degradation, DNA repair, transcription and signal transduction is particular important [1]. Recently, pupylation has been identified as the first post-translational protein-to-protein modification in prokaryotes [2,3]. Similar to ubiquitin, prokaryotic ubiquitin-like protein (Pup) attaches to specific lysine residues of substrate proteins by forming isopeptide bonds to target the proteins for proteasomal degradation [2,3].

Although ubiquitylation and pupylation are functional analogues, the enzymology of ubiquitylation and pupylation is different. In contrast to the three-step reaction of ubiquitylation, pupylation requires only two steps that only two enzymes are involved in pupylation. First, the C-terminal glutamine of Pup is deamidated to glutamine by deamidase of Pup (Dop) [4]. Subsequently, proteasome accessory factor A (PafA) attaches the deamidated Pup to specific lysine residues of substrate proteins [5].

The identification of pupylated proteins and pupylation sites can provide insights into the substrate specificity and functions of pupylation. Recently, large-scale proteomics technology has been applied to identify pupylated proteins and pupylation sites [6-9]. As the number of identified pupylated proteins and sites grows, a structured and searchable database of pupylated proteins and pupylation sites is desirable for further analyzing substrate specificity and functions of pupylated proteins and developing prediction methods for pupylation sites. For this purpose, the freely accessible database named PupDB integrating information of pupylated proteins and pupylation sites, protein structures, functional annotations and tools for browses, searches and interactive displays of protein structures and pupylation sites was constructed.

## Construction and content

The PupDB database is implemented using MySQL Server Edition 5.1. The PupDB website is publicly available at http://cwtung.kmu.edu.tw/pupdb. The web interface and all functions are implemented using PHP and Perl languages. The software of Google Chart Tools [10] is utilized to make sortable tables.

### Database content

Two kinds of proteins included in PupDB are pupylated proteins and candidate pupylated proteins. All proteins are collected from four large-scale proteomics studies [6-9]. Proteins with experimentally identified pupylation sites are annotated as pupylated proteins. Candidate pupylated proteins are experimentally identified proteins whose pupylation sites are still unknown.

**Figure 1:**
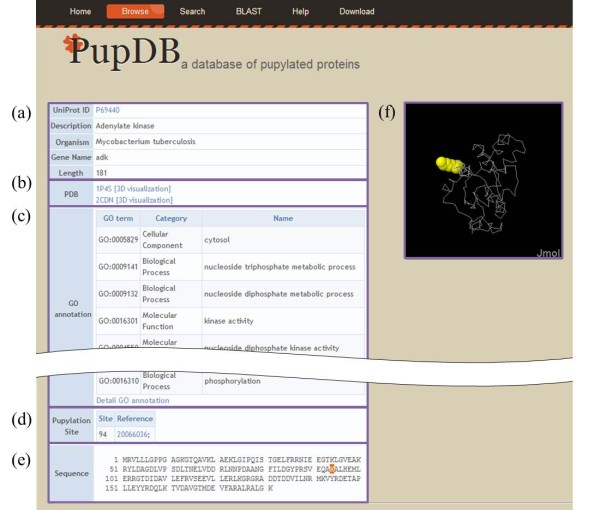
**Content of a typical PupDB entry**. (**a**) Basic information. (**b**) Structure. (**c**) GO annotation. (**d**) Pupylation site. (**e**) Sequence. (**f**) 3D structure.

Redundant proteins are removed from PupDB by using CD-HIT [11,12] with a sequence identity threshold of 98%. Currently, PupDB contains 182 pupylated proteins with 215 known pupylation sites and 1,123 candidate pupylated proteins. All proteins belong to three organisms of *Mycobacterium smegmatis*, *Mycobacterium tuberculosis *and *Escherichia coli*. For each protein, the corresponding information consists of six major parts of basic information, PDB ID, gene ontology (GO) annotation, pupylation site, protein sequence and structure as shown in Figure 1. PupDB will be regularly updated with additional data and corrections and analytical tools. Researchers are encouraged to contribute their data and suggestions to PupDB.

### Annotations

As shown in Figure 1a, the first part of basic information includes the UniProt AC, description, gene name, organism and sequence length. For further information of protein annotations, PupDB provides links to the corresponding entries of UniProt database [13]. Also, structure information including PDB (Protein Data Bank) ID and hyperlinks to the PDB database [14] is provided in the second part (Figure 1b). The visualization of pupylation sites in a protein structure can provide helpful information for analysis. The protein 3D structure and associated pupylation sites can be viewed in PupDB by clicking the link of '3D visualization'. The java applet-based program Jmol [15] is utilized for interactive displays of protein structure (Figure 1f). The UniProt protein accession numbers and PDB IDs are obtained by using the ID mapping function of UniProt. Currently, there are 766 PDB structures associated with 294 PupDB entries.

The GO annotations [16] can give useful information of molecular function, cellular component and biological process. For a given protein, the corresponding GO annotations can be extracted by using its UniProt accession number. Figure 1c shows the third part of GO annotations for protein P69440. Further GO information can be accessed by clicking the hyperlink of 'Detailed GO annotation' that links to the corresponding entry of QuickGO [17].

The fourth part of pupylation sites includes pupylation sites and corresponding references for pupylated proteins (Figure 1d). References are represented as PubMed IDs with hyperlinks to PubMed database [18]. Instead of showing only references for a candidate pupylated protein whose pupylation sites are still unknown, PupDB highlights pupylation sites in both sequence and structure of a pupylated protein for visualization as shown in Figure 1e and 1f, respectively.

## Utility and discussion

**Figure 2:**
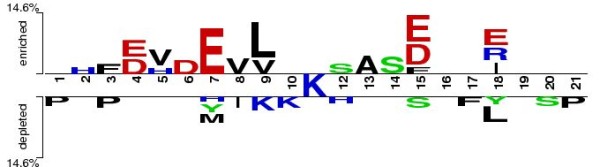
**Two-Sample Logo representation of over- (upper half) and underrepresented (lower half) residues surrounding pupylation sites**.

PupDB is a database of pupylated proteins and pupylation sites aiming to provide an easily accessible web service for the analysis of pupylated proteins. The analysis of pupylated proteins in PupDB can provide better insights into the specificity of pupylation. For example, Two Sample Logo [19] can be utilized to graphically analyze over- and underrepresented residues surrounding pupylation sites as shown in Figure 2.

Hyperlinks to major protein, structure and annotation databases are provided for accessing related information. Four useful tools are constructed and integrated into PupDB to provide functions of browses, keyword searches, sequence similarity searches and interactive displays of protein structures. The functions of the integrated tools are introduced in the follows.

### Browse tool

**Figure 3:**
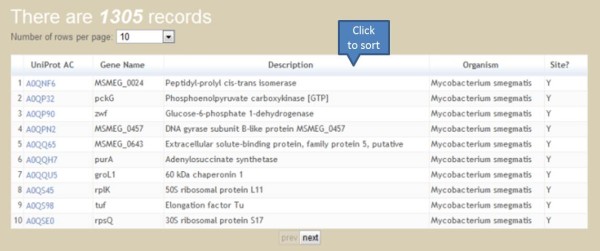
**Browse tool**.

Users can browse PupDB by selecting the 'Browse' option. All proteins will be shown in a sortable table. The entry with 'Y' in the field of 'Site' is a pupylated protein. Otherwise, it is a candidate pupylated protein with 'N' in the field of 'Site'. By clicking the caption of a specific column in a sortable table, the output table will be sorted according to data of the selected column. Furthermore, users can specify the number of rows shown per page (Figure 3).

### Search and BLAST tools

**Figure 4:**
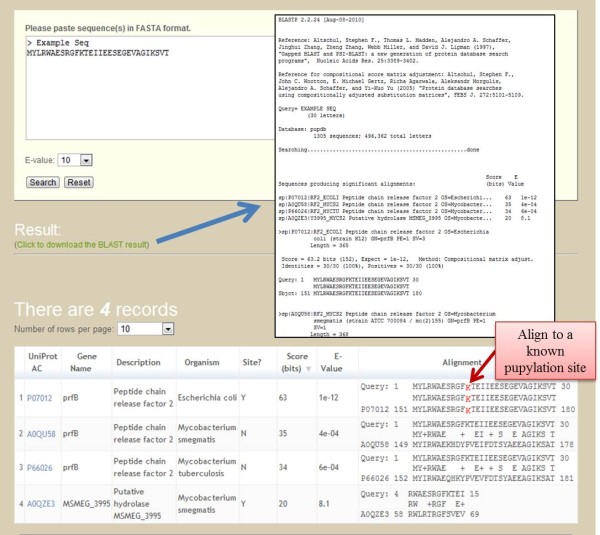
**BLAST tool**.

For retrieving entries of interest, PupDB provides two search tools of keyword and similarity searches. 1) The tool of keyword search can be accessed by selecting the 'Search' option. There are six fields for searching PupDB including description, UniProt AC, gene name, organism, protein type and protein with structure. By entering keywords for any one or combination of the fields, PupDB will return search results as a sortable table according to the user input keywords. 2) Users can enter a protein sequence of interest in FASTA format to perform a BLAST [20] search against PupDB to fetch entries with a user-defined threshold of E-value. The BLAST tool can serve as a potentially useful tool for predicting promising pupylation sites by sequence similarity. In addition to the protein information, three additional columns of scores, E-values and alignments obtained from the BLAST search are included in the output sortable table. The detailed information of BLAST sequence alignment can be downloaded by clicking the download link. Figure 4 shows an example of BLAST search. In the query sequence, lysines aligned to known pupylation sites will be marked in red color. Users can submit proteins in other organisms to predict pupylation sites.

### Interactive tool for protein structure

PupDB incorporates the Jmol applet of latest version 12.2 for interactive displays of protein structures. By default, PupDB represents protein structures and pupylation sites in grey and yellow colors, respectively. Users can either use the user interface or scripting console to manipulate protein structures.

## Conclusions

The PupDB database is a comprehensive repository of pupylated proteins and pupylation sites with a web-based user interface. The built-in tools for browses, searches and interactive displays of protein structures and pupylation sites make PupDB a useful resource for further analyzing the substrate specificity, identifying pupylated proteins in other organisms and developing computational tools for predicting pupylation sites. In addition to the graphical analysis using two-sample logos, advanced machine learning methods such as string kernels [21] can also be utilized to further analyze the specificity of pupylation. The exported dataset of pupylated proteins is downloadable at PupDB.

Post-translational modification databases serve as good data source for developing prediction tools. For example, the construction of UbiPred [22] for predicting ubiquitylation sites is based on dataset of UbiProt [23]. Although a predictor GPS-PUP [24] is available for predicting pupylation sites, PupDB with 215 pupylation sites can be utilized to further improve GPS-PUP trained on only 127 pupylation sites. Future works are two-fold. First, the development and integration of prediction tools based on the dataset of PupDB would be useful for analyzing and predicting pupylation sites. Second, the incorporation of orthology relationships and locations of functional domains can largely improve PupDB.

## Availability and requirements

The PupDB is freely available at http://cwtung.kmu.edu.tw/pupdb. The website has been tested with browsers of Safari, Opera, Internet Explorer 7 or later, Firefox and Google Chrome. The Java Runtime Environment (JRE) is required for interactive displays of protein 3D structures by Jmol.

## Competing interests

The author declares that they have no competing interests.

## Authors' contributions

CWT designed and implemented the database, performed the analysis and wrote the manuscript.
